# Assessment of a biofluid mechanics-based model for calculating portal pressure in canines

**DOI:** 10.1186/s12917-020-02478-1

**Published:** 2020-08-26

**Authors:** Jia-Yun Lin, Chi-Hao Zhang, Lei Zheng, Chen-Lu Song, Wen-Sheng Deng, Yi-Ming Zhu, Li Zheng, Li-Zhong Wu, Long-Ci Sun, Meng Luo

**Affiliations:** 1 Department of General Surgery, Shanghai Ninth People’s Hospital, School of Medicine, Shanghai Jiao Tong University, No. 639 Zhizaoju Road, Shanghai, 200011 China; 2 Department of Plastic Surgery, Shanghai Ninth People’s Hospital, School of Medicine, Shanghai Jiao Tong University, No. 639 Zhizaoju Road, Shanghai, 200011 China; 3 Department of Ultrasound, Shanghai Baoshan Integrated Traditional Chinese and Western Medicine Hospital, No. 181 Youyi Road, Shanghai, 201900 China; 4 Department of Radiation, Shanghai Ninth People’s Hospital, School of Medicine, Shanghai Jiao Tong University, No. 639 Zhizaoju Road, Shanghai, 200011 China; 5 Department of Gastrointestinal Surgery, Renji Hospital, School of Medicine, Shanghai Jiao Tong University, No.160 Pujian Road, Shanghai, 200127 China

**Keywords:** Biofluid mechanics, Non-invasive, Portal hypertension, Portal pressure

## Abstract

**Background:**

Portal hypertension is a severe complication caused by various chronic liver diseases. The standard methods for detecting portal hypertension (hepatic venous pressure gradient and free portal pressure) are available in only a few hospitals due to their technical difficulty and invasiveness; thus, non-invasive measuring methods are needed. This study aimed to establish and assess a novel model to calculate free portal pressure based on biofluid mechanics.

**Result:**

Comparison of each dog’s virtual and actual free portal pressure showed that a biofluid mechanics-based model could accurately predict free portal pressure (mean difference: -0.220, 95% CI: − 0.738 to 0.298; upper limit of agreement: 2.24, 95% CI: 1.34 to 3.14; lower limit of agreement: -2.68, 95% CI: − 3.58 to − 1.78; intraclass correlation coefficient: 0.98, 95% CI: 0.96 to 0.99; concordance correlation coefficient: 0.97, 95% CI: 0.93 to 0.99) and had a high AUC (0.984, 95% CI: 0.834 to 1.000), sensitivity (92.3, 95% CI: 64.0 to 99.8), specificity (91.7, 95% CI: 61.5 to 99.8), positive likelihood ratio (11.1, 95% CI: 1.7 to 72.8), and low negative likelihood ratio (0.08, 95% CI: 0.01 to 0.6) for detecting portal hypertension.

**Conclusions:**

Our study suggests that the biofluid mechanics-based model was able to accurately predict free portal pressure and detect portal hypertension in canines. With further research and validation, this model might be applicable for calculating human portal pressure, detecting portal hypertensive patients, and evaluating disease progression and treatment efficacy.

## Background

Portal hypertension is known as a severe disease with a poor outcome. It can threaten people’s lives if the gastroesophageal varices rupture and are not treated in time [1, 2]. Portal pressure measurement is of great importance, because it is an effective method for evaluating liver disease progression [3]. However, the application of the hepatic venous pressure gradient (HVPG) and free portal pressure (FPP), the standard methods for detecting portal hypertension, are restricted due to technical difficulty and invasiveness [4–6]. Although there have been studies of non-invasive portal hypertension assessment methods, including clinical examination, ultrasound, elastography, CT and magnetic resonance imaging [7, 8], few were proven to be reliable. Recently, Yang [9–11] established three-dimensional hepatic portal venous models from CT images to evaluate the severity of cirrhotic patients, which showed good performance; however, the models were neither introduced in detail nor validated in a large population. Therefore, an accurate and non-invasive portal pressure measuring method is needed and would be useful in the diagnosis and evaluation of portal hypertension.

Biofluid mechanics is the study of biological flow mechanisms and the inter-relationships with physiological and pathological processes using the fundamental principles of fluid mechanics [12]. Using biofluid mechanics, cardiologists succeeded in calculating fractional flow reserve, the standard assessment of the haemodynamics of coronary stenoses [13, 14]. This motivated us to establish a non-invasive method for accurately calculating FPP.

The aim of this study was to establish and assess a biofluid mechanics-based model for predicting FPP and detecting portal hypertension in canines.

## Results

**Fig. 1:**
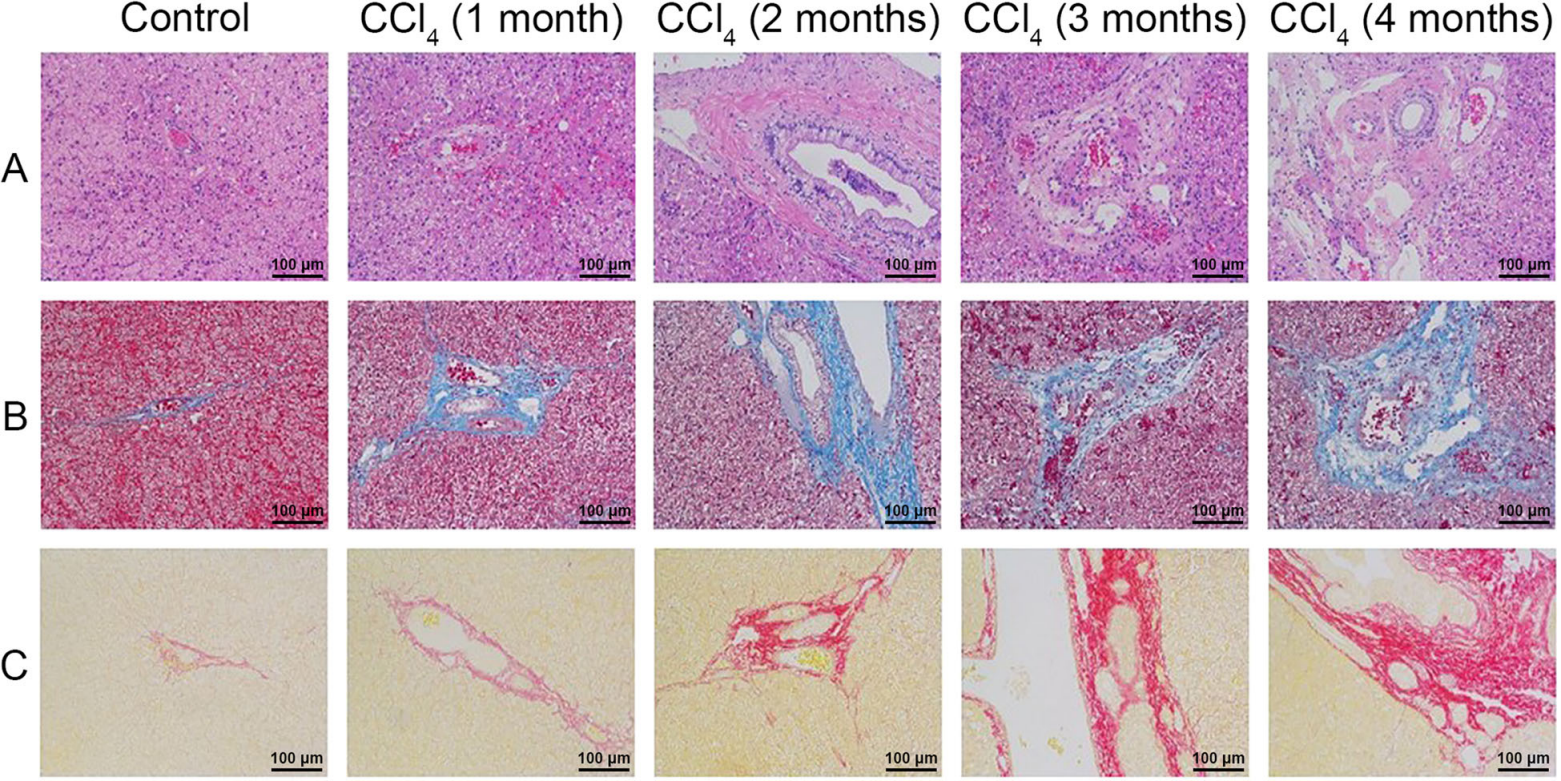
Liver fibrogenesis of canines with a continuous modelling time. **a** Hematoxylin-eosin staining. **b** Masson’s trichrome staining. **c** Sirius red staining. CCl_4_: carbon tetrachloride

To validate canine portal hypertensive models, we focused on canine liver fibrogenesis (Fig. 1), liver function, FPP (Table 1), as well as blood vessel diameters (Table 2) and blood flow velocity (Table 3) of their portal venous system. We found that canines from CCl_4_-treated groups developed liver fibrogenesis, had lower blood flow velocity, wider blood vessel diameters, and higher liver function and FPP values as compared with those from a control group. These results proved the success of our canine portal hypertensive model.

**Table 1:** Liver function and FPP of canines

Parameters	Control	CCl_4_ (1 month)	CCl_4_ (2 months)	CCl_4_ (3 months)	CCl_4_ (4 months)
Total bilirubin (μmol/L)	2.2 ± 0.9	4.6 ± 1.0*	6.5 ± 0.7*	5.2 ± 2.5	7.3 ± 0.8*
Direct bilirubin (μmol/L)	0.5 ± 0.3	0.5 ± 2.9	2.3 ± 0.3*	2.3 ± 2.3	3.4 ± 1.3*
Indirect bilirubin (μmol/L)	1.7 ± 0.7	4.0 ± 0.9*	4.2 ± 0.6*	3.0 ± 0.3*	3.9 ± 0.8*
Alanine aminotransferase (U/L)	21 ± 8	81 ± 47*	481 ± 178*	78 ± 46	366 ± 110*
Aspartate transaminase (U/L)	28 ± 9	36 ± 8	50 ± 20	29 ± 12	58 ± 13*
free portal pressure (mmHg)	6.6 ± 1.1	10.0 ± 1.6*	13.6 ± 1.5*	15.6 ± 4.0*	19.2 ± 1.9*

Note. Values are presented as the M ± SD; * meaning *P* < 0.05 vs. Control group; *CCl*_*4*_ carbon tetrachloride

**Table 2:** Blood vessel diameters (mm) of canines’ portal venous system

Vessel (mm)	Control	CCl_4_ (1 month)	CCl_4_ (2 months)	CCl_4_ (3 months)	CCl_4_ (4 months)
Portal vein	4.5 ± 0.4	7.2 ± 0.6*	7.7 ± 1.2*	8.2 ± 1.0*	9.5 ± 1.3*
Left portal vein	3.4 ± 0.3	4.9 ± 0.7*	5.9 ± 0.9*	6.2 ± 1.4*	7.6 ± 1.2*
Right portal vein	4.2 ± 0.2	4.9 ± 0.4*	5.2 ± 0.6*	5.8 ± 0.6*	6.8 ± 1.2*
Splenic vein	1.8 ± 0.2	3.4 ± 0.5*	4.0 ± 0.5*	4.5 ± 0.7*	4.9 ± 0.8*
Superior mesenteric vein	3.1 ± 0.3	4.1 ± 0.2*	4.8 ± 0.5*	5.8 ± 0.7*	6.0 ± 0.6*
Inferior mesenteric vein	2.0 ± 0.1	2.4 ± 0.3*	2.9 ± 0.3*	3.5 ± 0.4*	3.8 ± 0.4*

Note. Values are presented as the M ± SD; * meaning *P* < 0.05 vs. Control group; *CCl*_*4*_ carbon tetrachloride

**Table 3:** Blood flow velocity (cm/s) of canines’ portal venous system

Vessel (cm/s)	Control	CCl_4_ (1 month)	CCl_4_ (2 months)	CCl_4_ (3 months)	CCl_4_ (4 months)
Portal vein	33.8 ± 8.7	30.6 ± 6.3	24.8 ± 3.7	25.1 ± 1.5	22.0 ± 1.6*
Left portal vein	22.6 ± 4.4	23.2 ± 2.9	13.7 ± 3.4*	11.4 ± 2.3*	9.7 ± 0.5*
Right portal vein	24.2 ± 4.2	25.4 ± 1.3	14.5 ± 4.9	11.7 ± 2.1*	10.6 ± 0.6*
Splenic vein	16.5 ± 2.3	16.5 ± 2.0	13.8 ± 2.9	18.5 ± 6.5	13.7 ± 2.2*
Superior mesenteric vein	27.3 ± 4.3	27.5 ± 4.5	18.0 ± 0.9*	17.7 ± 2.3*	16.5 ± 3.0*
Inferior mesenteric vein	15.0 ± 1.6	13.0 ± 1.6	10.7 ± 1.8*	11.1 ± 1.1*	10.3 ± 0.5*

Note. Values are presented as the M ± SD; * meaning *P* < 0.05 vs. Control group; *CCl*_*4*_ carbon tetrachloride

**Fig. 2:**
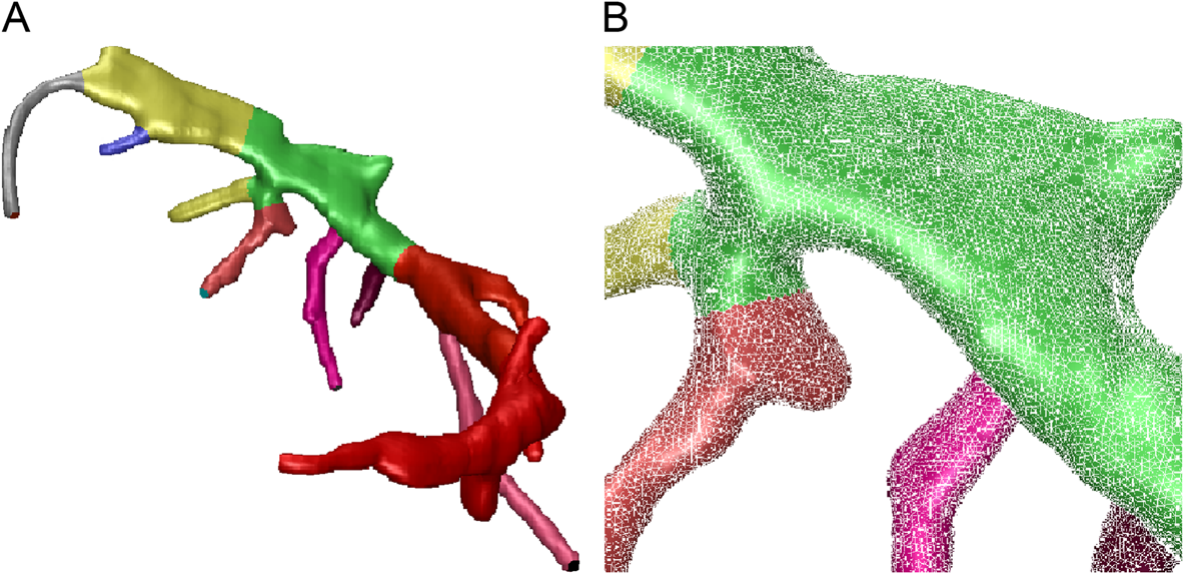
**a** The simulation model of the portal venous system, different colours representing different parts. **b** The body meshes of the simulation model

**Fig. 3:**
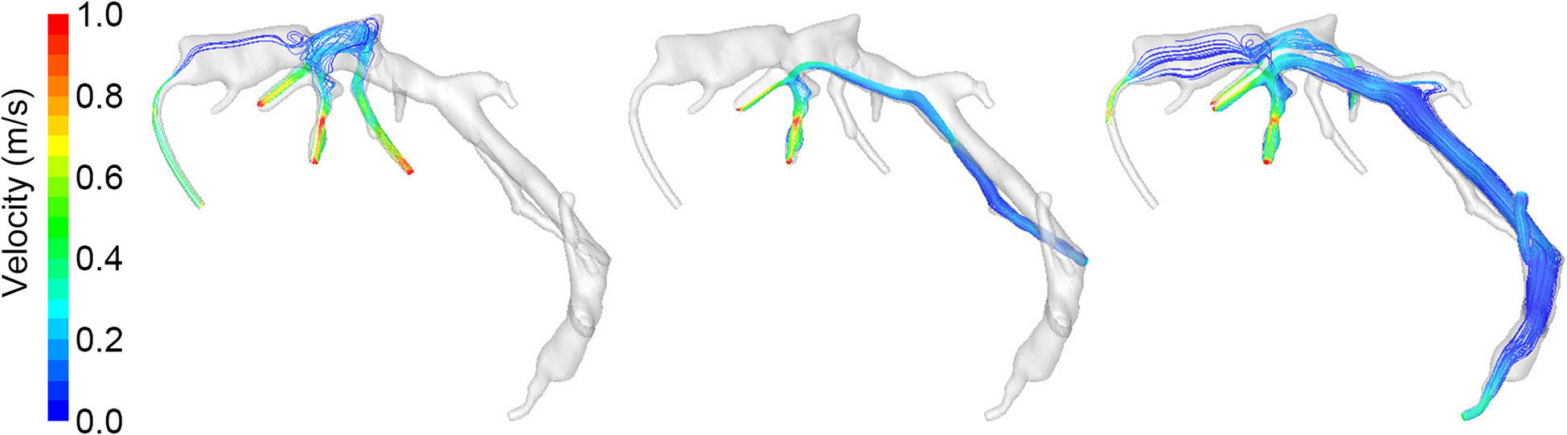
The blood flow simulation of the portal venous system

**Fig. 4:**
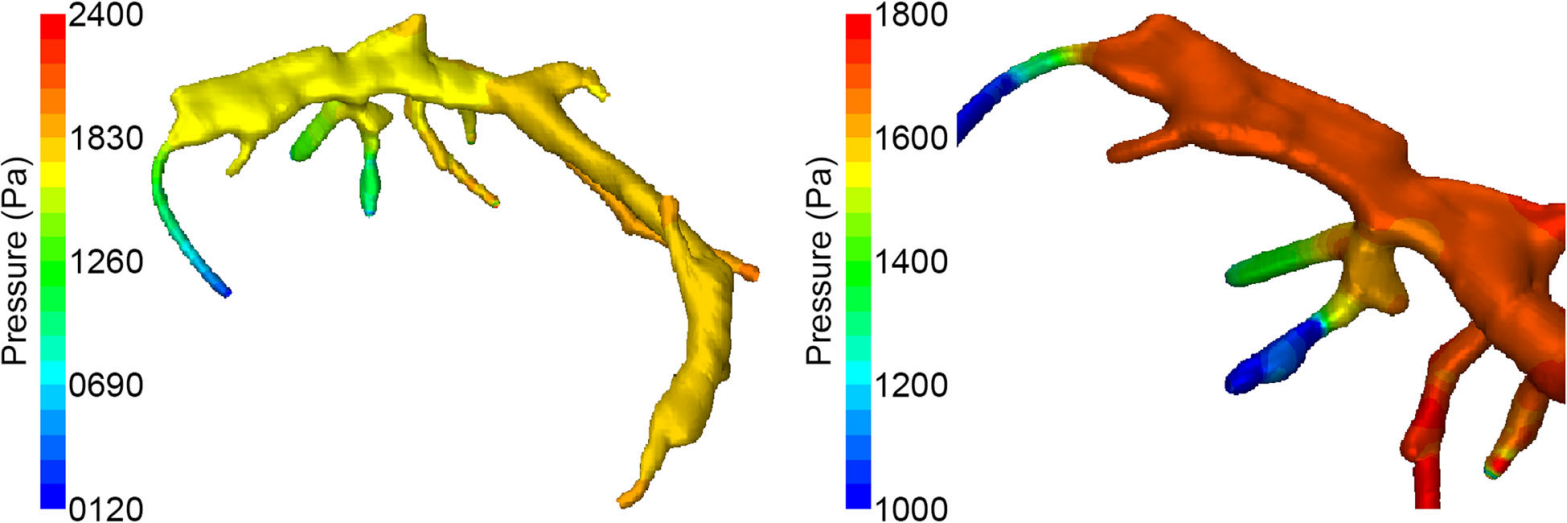
The blood pressure (Pa) of the portal venous system

We imported canine abdominal CT images into the IQQA-Liver system to produce a three-dimensional portal venous model, in which the portal vein and its main branches, including the left and right portal vein, the splenic vein, the superior mesenteric vein, and the inferior mesenteric vein, were visualized precisely. We used FLUENT software to divide the model (one case is shown in Fig. 2a), mesh the model surfaces into triangular surface grids and create the body meshes accordingly (one case is shown in Fig. 2b). Blood density and overall viscosity were used as the properties of the blood. The “pressure outlet boundary conditions” module was used for the portal vein and the “velocity inlet boundary conditions” module was used for each inlet and outlet branch. The blood flow velocity at the boundaries of each branch was calculated (formula 1–2). FLUENT software was used to solve the Navier-Stokes equations (formula 3–8), simulate the blood flow within the portal venous system (one case is shown in Fig. 3), and obtain the virtual free portal pressure (vFPP) (one case is shown in Fig. 4).

**Fig. 5:**
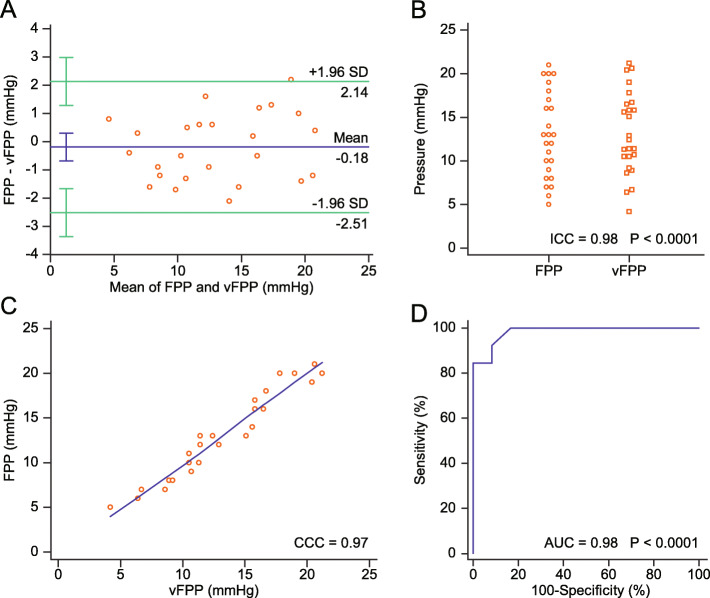
The numeric correlation between vFPP and FPP and the diagnostic performance of vFPP. **a** Bland and Altman’s limits of agreement analysis. **b** Intraclass correlation coefficient (ICC). **c** Lin’s concordance correlation coefficient (CCC). **d** Receiver Operating Characteristic (ROC) curve and Area under the ROC curve (AUC) of vFPP. FPP: free portal pressure; vFPP: virtual free portal pressure

To assess the numeric correlation between vFPP and FPP, we performed Bland and Altman’s limits of agreement analysis (Fig. 5a), the intraclass correlation coefficient (Fig. 5b), and Lin’s concordance correlation coefficient (Fig. 5c) between FPP and vFPP. For Bland and Altman’s limits of agreement analysis, the mean of the difference was − 0.220 (95% CI: − 0.738 to 0.298); the upper limit of agreement was 2.24 (95% CI: 1.34 to 3.14); the lower limit of agreement was − 2.68 (95% CI: − 3.58 to − 1.78). The intraclass correlation coefficient was 0.98 (95% CI: 0.96 to 0.99, *P* < 0.0001). The concordance correlation coefficient was 0.97 (95% CI: 0.93 to 0.99). These results showed that the vFPP model provided a good prediction of FPP.

To evaluate the performance of vFPP in the diagnostics of portal hypertension, we carried out receiver operating characteristic (ROC) curve analysis (Fig. 5d). The area under the ROC curve (AUC) was 0.984 (95% CI: 0.834 to 1.000, *P* < 0.0001). We selected vFPP = 12 mmHg as the criterion value. The sensitivity was 92.3 (95% CI: 64.0 to 99.8); the specificity was 91.7 (95% CI: 61.5 to 99.8); the positive likelihood ratio was 11.1 (95% CI: 1.7 to 72.8); the negative likelihood ratio was 0.08 (95% CI: 0.01 to 0.6).

All canines survived and had no important adverse events during the experiment.

## Discussion

Portal hypertension is a life-threatening disease. Portal pressure measurement is of great importance because it is the standard method for detecting portal hypertension. Moreover, portal pressure strongly correlates with severe complications, such as hepatocellular carcinomas, gastro-oesophageal variceal haemorrhaging, hepatic encephalopathy, and ascites [15]. In this study, we performed canine experiments to evaluate a biofluid mechanics-based model for calculating vFPP and detecting portal hypertension. Both FPP and HVPG are the standard methods for detecting portal hypertension [1, 2], however, we used only FPP as a reference because canines’ FPP can be easily measured, and balloon-tipped catheters for canine HVPG measurements were unavailable due to the narrowness of the canine vessels.

The IQQA system, the FLUENT software, and the Navier-Stokes equations have been widely used to reconstruct blood vessels, simulate blood flow, and precisely calculate the haemodynamics [16–24]. In the portal venous system, due to the relatively tiny size of blood cells (compared with the size of the vessels) and the steadily fast blood flow, the blood within could be modelled as an incompressible Newtonian fluid; therefore, it is appropriate to apply the IQQA system, the FLUENT software and the Navier-Stokes equations to our study [25, 26]. Based on previous studies, we built the vFPP calculation model using IQQA and FLUENT software to reconstruct canine portal venous system, simulate the blood flow, and calculate the vFPP by the Navier-Stokes equations. After assessing the model by Bland and Altman’s limits of agreement analysis, the intraclass correlation coefficient, Lin’s concordance correlation coefficient and ROC curve analysis, we showed that this model was able to predict FPP and diagnose portal hypertension accurately.

Iranmanesh and Liu also established models for detecting portal hypertensive patients, which showed good diagnostic performance with high sensitivity and specificity [27, 28]. Although their works were able to detect patients suffering from clinically significant portal hypertension (HVPG greater than 10 mmHg) well, their models were not suitable for mild or moderate portal hypertensive patients whose HVPG were less than 10 mmHg. Therefore, we administered CCl_4_ to dogs for 1, 2, 3 or 4 months to represent different stages of portal hypertension. Our result showed that our model was suitable for not only severe but also mild portal hypertensive and even normal canine functionality. Moreover, our model was able to predict the vFPP value, which was another advantage over previous models. We used five time points (0, 1, 2, 3, 4 months), because there were few references reporting the adverse events for this method. We were concerned that some canines might be unable to survive 4 months of continuous injection of CCl_4_ and planned to terminate CCl_4_ injection if two dogs from the earliest group died. For example, if two dogs had lived for only 2 months during continuous injection of CCl_4_, then we would have terminated CCl_4_ injection at that time point; therefore, there would have been only three groups left: Control, CCl_4_ (1 month), and CCl_4_ (2 months). However, if we had designed only three time points (0, 2, 4 months) in this study, then in this situation, there would have been only two groups left: Control and CCl_4_ (2 months), which would have been insufficient to reflect different stages of cirrhosis and portal hypertension.

Our non-invasive methods could calculate vFPP from CT, blood tests, and Doppler ultrasound results. Similarly, we could use these methods to simulate the blood flow of the inferior vena cava and hepatic veins, and calculate the virtual hepatic venous pressure (vHVP) and virtual HVPG (the difference between vFPP and vHVP). With further research and validation, this model might be applicable for calculating human vFPP and virtual HVPG, detecting portal hypertensive patients, and evaluating disease progression and treatment efficacy.

There are some limitations in this study. Firstly, although we demonstrated a high correlation between vFPP and FPP, this result was acquired from canines, not humans. We chose beagles as experimental animals because they have a moderate body size and a digestive system similar to humans. It is also easy to perform examinations on them. In addition, the methods and results of this study might be applied to humans more appropriately. After this study, we applied this model to several portal hypertensive patients who underwent portosystemic shunts or splenectomy with periesophagogastric devascularisation and found similar results (unpublished observations). We are currently carrying out a prospective, randomised, non-controlled, multicentre trial (trial registration number: NCT03470389) to further validate this model in humans [29]. Secondly, the intra-abdominal pressure might have changed after general anaesthesia and abdominal incision, which might have influenced the FPP. Thirdly, the accuracy of the time-intensity curves of the Doppler ultrasound might have been affected by respiratory motion artefacts; this problem was partly alleviated by limiting the breathing extent of the canines.

## Conclusion

The non-invasive and biofluid mechanics-based model could accurately predict FPP and had high sensitivity and specificity for detecting portal hypertension in canines. With further research and validation, this model might be applicable for calculating human FPP, detecting patients with portal hypertension, and evaluating disease progressions and treatment efficacies.

## Methods

### Animal model

The canines used in this study were purchased from the laboratory animal department of Shanghai Jiagan Biotechnology Co., Ltd. as experimental animals. The study protocol was reviewed and approved by the Animal Care and Use Committee of Shanghai Ninth People’s Hospital, School of Medicine, Shanghai Jiao Tong University. All procedures were conducted according to the Animal Experimentation Guidelines of Shanghai Jiao Tong University. The study was performed on 25 adult male beagles (10.5 to 12.5 kg), which were caged with constant temperature (25 °C), humidity (60 ± 10%), and circadian-rhythmic lighting in the laboratory animal department of Shanghai Jiagan Biotechnology Co., Ltd. To reflect different stages of cirrhosis and portal hypertension, canine portal hypertensive models were induced by continuous subcutaneous injection of CCl_4_. The 25 dogs were divided randomly into five 5-member groups: Control, CCl_4_ (1 month), CCl_4_ (2 months), CCl_4_ (3 months), and CCl_4_ (4 months). Randomisation was based on a computer-generated random digits table. Each dog’s group was blind to the researchers responsible for histological staining, laboratory tests, Doppler ultrasound, CT, FPP measurement, and vFPP computation in order to prevent biases. The 20 dogs in the CCl_4_-treated groups began receiving CCl_4_ administration 1, 2, 3 or 4 months before the end of the study, so that all dogs reached the end of the study simultaneously. CCl_4_ was dissolved in olive oil (60% CCl_4_ and 40% olive oil) and injected subcutaneously in the dorsal area of the canines. This administration was repeated every 10 days at a dose of 1.0 to 1.3 ml/kg. Histological staining, laboratory tests, Doppler ultrasound, CT, FPP measurement, and vFPP computation were performed at the end of the study. All canines survived and continued to live in the laboratory animal department of Shanghai Jiagan Biotechnology Co., Ltd. after the study.

### Histological staining

Hepatic tissue was taken and fixed in 10% formalin and embedded in paraffin. The paraffin-embedded tissue was sectioned at 5 μm and then placed on slides, deparaffinized in xylene, hydrated in decreasing concentrations of ethanol, and washed in water. After hematoxylin-eosin staining, Masson’s trichrome staining, and Sirius red staining, the sections were examined under a microscope.

### Laboratory test

Each dog’s peripheral blood samples were taken from the small saphenous vein of the hind leg for blood viscosity and liver function tests. The blood density test was performed by weighing 1 millilitre of blood using an electronic balance. The blood density measurements were repeated at least three times and then averaged.

### Doppler ultrasound

Each canine underwent an abdominal Doppler ultrasound scan after an overnight fast and a venous injection of pentobarbital sodium (30 mg/kg) in accordance with previously published methods [30]. Each canine was in dorsal recumbency throughout the scan. The ultrasound specialists measured the inner diameters and the maximum blood flow velocity of the portal vein and its main branches, including the right branch of the portal vein, the left branch of the portal vein, the portal vein, the splenic vein, the superior mesenteric vein, and the inferior mesenteric vein. The 3–5 MHz Doppler ultrasound probes were used and the insonation angles were between 45° and 65°. Each measurement was repeated twice; both intra-observer variability and inter-observer variability were less than 10%.

### Computed tomography

Each dog was fixed in the supine position and underwent an abdominal contrast-enhanced CT after an overnight fast and a venous injection of pentobarbital sodium (30 mg/kg) in accordance with previously published methods [31, 32]. A non-ionic iodinated contrast agent (600 mg of iodine per kilogram of body weight, 300 mg of iodine per ml, 5 ml per second) was injected. The arterial phase imaging began 10 s after the beginning of the intravenous contrast injection, and portal phase imaging began 30 to 40 s after the beginning of the intravenous contrast injection.

### FPP measurement

Each canine’s FPP was measured after general anaesthesia. An abdominal midline incision was made, exposing the right gastroepiploic vein, and a pressure sensor-connected catheter was inserted into the portal vein through the right gastroepiploic vein. The FPP was recorded by a physiological signal acquisition system. The right atrium pressure was defined as the zero reference point.

### vFPP computation

The simulation model of the canine portal venous system, which was created from canine CT images by the IQQA-Liver system version 2.0 (EDDA Technology, Inc., USA), was imported into the Fluent software version 6.3 (ANSYS, Inc., USA). The model was divided into different parts, with each inlet and outlet branch identified as a separate part. The model surface was then meshed into 0.2–1.0 mm triangular surface grids and the body meshes were created accordingly. The laminar viscous model was used. The material type was set to fluid, blood density and overall viscosity were used as the properties of the fluid. The pressure outlet boundary conditions module was used for the portal vein, with the following parameters: backflow reference frame: absolute; gauge pressure: 0; backflow direction specification method: normal to boundary; radial equilibrium pressure distribution: disabled; average pressure specification: disabled; target mass flow rate: disabled. The velocity inlet boundary conditions module was used for each inlet and outlet branch, with the following parameters: velocity specification method: magnitude, normal to boundary; reference frame: absolute; supersonic/initial gauge pressure: 0. The velocity magnitude value was set to the blood flow velocity at the boundaries of each branch, which was calculated according to the inner diameter, blood flow velocity and direction measured by Doppler ultrasound, the inner diameter at the boundaries obtained from the simulation model, and the principle of mass conservation. The equations are as follows:

1 $$ \mathrm{Q}=\mathrm{Av}=\frac{1}{4}{\uppi \mathrm{d}}_{\mathrm{b}}^2{\mathrm{v}}_{\mathrm{b}}=\frac{1}{4}{\uppi \mathrm{d}}_{\mathrm{us}}^2{\mathrm{v}}_{\mathrm{us}} $$

2 $$ {\mathrm{v}}_{\mathrm{b}}=\frac{{\mathrm{d}}_{\mathrm{us}}^2}{{\mathrm{d}}_{\mathrm{b}}^2}{\mathrm{v}}_{\mathrm{us}} $$

Q: volume flow rate; A: cross-sectional area; v: velocity; d_b_: inner diameter at the boundaries; v_b_: velocity at the boundaries; d_us_: inner diameter measured by Doppler ultrasound; v_us_: velocity measured by Doppler ultrasound.

**Fig. 6:**
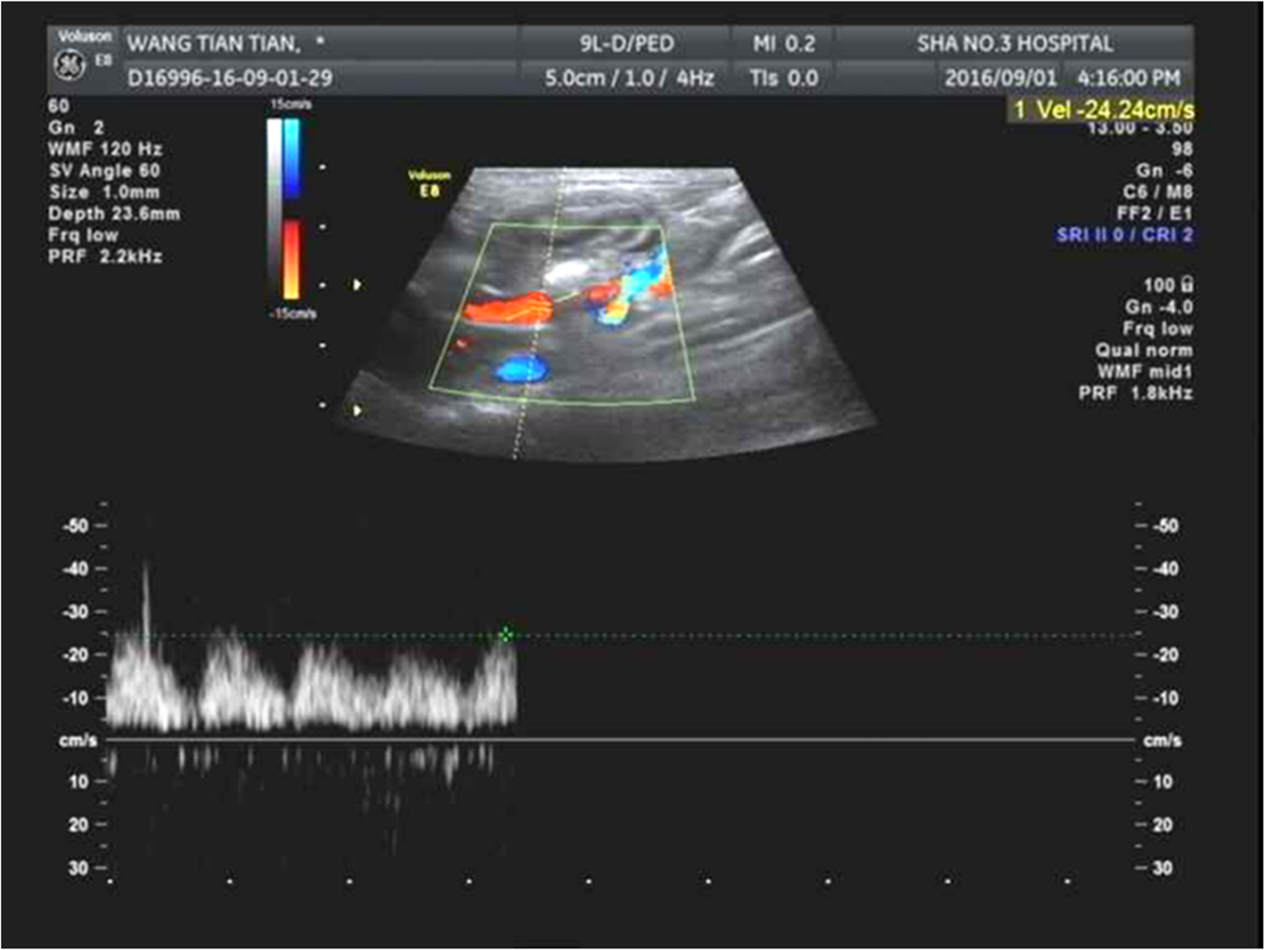
The Doppler ultrasound image of the portal venous system

According to Doppler ultrasound images (one case is shown in Fig. 6), the mean blood flow velocity approximately equals 0.7 times the maximum blood flow velocity. Since the blood within the portal venous system can be assumed to be an incompressible Newtonian fluid, blood flow was modelled by the Navier-Stokes equations as follows:

The mass conservation equation:

3 $$ \frac{\mathrm{\partial \uprho }}{\mathrm{\partial t}}=\nabla \cdot \left(\uprho \overrightarrow{\mathrm{v}}\right)={\mathrm{S}}_{\mathrm{m}} $$

ρ: density; t: time; S_m_: mass added to the continuous phase.

For axisymmetric geometries, the mass conservation equation can be written as follows:

4 $$ \frac{\mathrm{\partial \uprho }}{\mathrm{\partial t}}+\frac{\partial }{\mathrm{\partial x}}\left(\uprho {\mathrm{v}}_{\mathrm{x}}\right)+\frac{\partial }{\mathrm{\partial r}}\left(\uprho {\mathrm{v}}_{\mathrm{r}}\right)+\frac{\uprho {\mathrm{v}}_{\mathrm{r}}}{\mathrm{r}}={\mathrm{S}}_{\mathrm{m}} $$

x: axial coordinate; r: radial coordinate; v_x_: axial velocity; v_r_: radial velocity.

The momentum conservation equation:

5 $$ \frac{\partial }{\mathrm{\partial t}}\left(\uprho \overrightarrow{\mathrm{v}}\right)+\nabla \cdot \left(\uprho \overrightarrow{\mathrm{v}}\overrightarrow{\mathrm{v}}\right)=-\nabla \mathrm{p}+\nabla \cdot \left(\overline{\overline{\uptau}}\right)+\uprho \overrightarrow{\mathrm{g}}+\overrightarrow{\mathrm{F}} $$

p: static pressure; $\overline{\overline{\uptau}}$: stress tensor; $\mathrm{p}\overrightarrow{\mathrm{g}}$: gravitational body force; $\overrightarrow{\mathrm{F}}$: external body force.

$\overline{\overline{\uptau}}$, the stress tensor, can be written as follows:

6 $$ \overline{\overline{\uptau}}=\upmu \left[\left(\nabla \overrightarrow{\mathrm{v}}+\nabla {\overrightarrow{\mathrm{v}}}^{\mathrm{T}}\right)-\frac{2}{3}\nabla \cdot \overrightarrow{\mathrm{v}}\mathrm{I}\right] $$

μ: molecular viscosity; I: unit tensor; T: temperature.

The energy conservation equation:

7 $$ \frac{\partial }{\mathrm{\partial t}}\left(\uprho \mathrm{E}\right)+\nabla \cdot \left(\overrightarrow{\mathrm{v}}\left(\uprho \mathrm{E}+\mathrm{p}\right)\right)=\nabla \cdot \left({\mathrm{k}}_{\mathrm{eff}}\nabla \mathrm{T}\hbox{-} {\Sigma}_{\mathrm{j}}{\mathrm{h}}_{\mathrm{j}}{\overrightarrow{\mathrm{J}}}_{\mathrm{j}}+\left({\overline{\overline{\uptau}}}_{\mathrm{eff}}\cdot \overrightarrow{\mathrm{v}}\right)\right)+{\mathrm{S}}_{\mathrm{h}} $$

E: total energy of fluid; k_eff_: effective conductive coefficient; h: enthalpy; J: diffusion flux; S_h_: volumetric heat sources.

E, the total energy of fluid, can be written as follows:

8 $$ \mathrm{E}=\mathrm{h}\hbox{-} \frac{\mathrm{p}}{\uprho}+\frac{{\mathrm{v}}^2}{2} $$

The FLUENT software was used to solve the Navier-Stokes equations, simulate the blood flow within the portal venous system, and obtain the pressure on each volume grid. The vFPP equals the pressure at the centre of the portal vein.

### Statistical analysis

Continuous variables were checked for normal distribution and summarized by either mean ± standard deviation or median and inter-quartile range, as appropriate. Comparison of continuous variables were performed using Student’s t-test or analysis of variance for normally distributed variables and the Mann-Whitney U test or the Kruskal-Wallis test for non-normally distributed variables as appropriate. The numeric correlation between FPP and vFPP was analysed by Bland and Altman’s limits of agreement analysis [33]. Bias was defined as the mean of the difference between FPP and vFPP. Upper and lower limits of agreement were defined as mean difference ± 1.96 standard deviation of the difference. The numeric correlation between FPP and vFPP was also analysed by the intraclass correlation coefficient and Lin’s concordance correlation coefficient. The diagnostic accuracy of vFPP for detecting portal hypertension (FPP greater than 12 mmHg) was assessed by ROC curve analysis, the sensitivity, the specificity, and the likelihood ratio. All tests of significance were at the 5% significance level. Analyses were performed using SPSS version 24.0 (IBM, USA) and MedCalc Statistical Software version 18.11 (MedCalc Software bvba, Belgium).

## Data Availability

The datasets used and/or analysed during the current study are available from the corresponding author on reasonable request.

## Abbreviations

CT: Computed tomography
CI: Confidence interval
HVPG: Hepatic venous pressure gradient
FPP: Free portal pressure
CCl_4_: Carbon tetrachloride
ROC: Receiver operating characteristic
AUC: Area under the ROC curve
ICC: Intraclass correlation coefficient
CCC: Concordance correlation coefficient
vFPP: Virtual free portal pressure
vHVP: Virtual hepatic venous pressure
